# An integrative review of research on gambling and domestic and family violence: Fresh perspectives to guide future research

**DOI:** 10.3389/fpsyg.2022.987379

**Published:** 2022-10-13

**Authors:** Nerilee Hing, Cathy O’Mullan, Lydia Mainey, Nancy Greer, Helen Breen

**Affiliations:** ^1^School of Health, Medical and Applied Sciences, Central Queensland University, Bundaberg, QLD, Australia; ^2^School of Nursing and Midwifery, Central Queensland University, Cairns, QLD, Australia; ^3^Faculty of Business and Law and Arts, Southern Cross University, Lismore, NSW, Australia

**Keywords:** intimate partner violence, domestic violence, gender-based violence, gambling harm, gambling disorder, problem gambling

## Abstract

This paper presents an integrative review of research on domestic and family violence (DFV), including intimate partner violence (IPV), experienced by victims and perpetrators with a gambling problem. It aims to review, critique, and synthesize research on this topic to generate fresh and alternative perspectives to guide future research. Based on a systematic search of the academic literature and a targeted search of gray literature, the paper summarizes findings from empirical studies pertaining to the prevalence of perpetration and victimization, characteristics of perpetrators and victims, and explanations for this violence. Based on this review, the paper suggests several potential improvements that can be considered in future studies. These include a shift from focusing on situational violence to also include coercive control; greater sensitivity in research design and interpretation to gender differences in experiences of violence; and the need to include economic abuse as a form of DFV/IPV. Adopting a public health lens is also recommended to broaden the research focus from victims and perpetrators to also consider contextual factors. In particular, gambling research should examine the contribution of gambling products, practices, environments, and marketing to DFV/IPV and how this might be ameliorated. While research to date has drawn much needed attention to the risks that gambling presents for DFV/IPV, this review provides some suggestions for future research so that it can provide more nuanced findings to inform policy and practice.

## Introduction

Harm from gambling extends beyond people who gamble to also diminish the health and wellbeing of significant others, including intimate partners and family members (Browne et al., 2016; Tulloch et al., 2021). One harm associated with gambling is domestic and family violence, with higher rates of perpetration and victimization found among individuals with a gambling problem (Afifi et al., 2010; Dowling et al., 2018; Roberts et al., 2018). Intimate partners are most commonly the victims and perpetrators of this violence, although violence related to gambling also occurs amongst other family members (Dowling et al., 2014; Bellringer et al., 2017; Palmer du Preez et al., 2018). Gambling research has sought to understand the prevalence of this violence, characteristics of perpetrators and victims, why this violence occurs, and how it is linked to gambling.

Intimate partner violence (IPV) refers to behavior by an intimate partner or ex-partner that causes physical, sexual, or psychological harm, including physical aggression, sexual coercion, psychological abuse, and controlling behaviors (World Health Organization [WHO], 2017). Domestic and family violence (DFV) is a broader term that refers to violence between family members, as well as violence between intimate partners. Definitions of DFV and IPV cover a wide range of behaviors. They commonly refer to physical and sexual violence, threats and intimidation, psychological and emotional abuse, and social and economic deprivation; unequal power whereby the perpetrator uses violence to dominate and control the victim; frequent and infrequent violence; severe and less severe violence; and impacts including fear, physical and psychological harm, and reduced quality of life for victims (Morgan and Chadwick, 2009).

Given the wide scope of abusive behaviors, researchers have distinguished between different types of DFV/IPV in seeking to understand the complexity of the phenomenon and in recognition that different motives drive different types of violence and their impacts. For example, a seminal conceptualization distinguished four types of violence (Johnson, 2005, 2010). *Situational violence* is characterized by specific conflicts that are situationally-provoked and that escalate to abuse in response to rising tensions or emotions, but are not connected to a general pattern of controlling behaviors (Johnson, 2005, 2010). In contrast, *intimate terrorism* is a pattern of coercive control, used mostly by men against a female partner, and characterized by intimidation, isolation, control, and assault to subordinate the victim and acquire privileges such as resources, sex, and personal service (Johnson, 2006, 2010; Stark, 2009). It is an instrumental pattern of oppression motivated by the desire to exercise power and control over the victim’s actions, relationships, and activities through micro-regulation of their everyday behaviors (Kimmel, 2002; Stark, 2009; Johnson, 2010; Tanha et al., 2010). *Violent resistance* by victims also occurs, motivated by self-defense, desperation, or a means to escape a violent perpetrator; while rare cases of *mutual violent control* occur where both parties are violent and controlling toward each other (Johnson, 2010). Research into DFV/IPV has become increasingly attuned to these different types of violence and their patterns, prevalence, and impacts on victims, providing more nuanced findings to inform primary, secondary, and tertiary interventions. Coercive control, in particular, continues to gain greater recognition in policy, practice, and regulation in attempts to prevent the most traumatizing, injurious, and lethal type of IPV.

With these issues in mind, this paper aims to (1) review research on the links between gambling and DFV/IPV, including prevalence, characteristics of victims and perpetrators, and explanations for gambling-related violence; and (2) advance some new perspectives to enable future gambling studies to provide more complete, accurate and nuanced findings to inform policy and practice.

## Methods

An integrative literature review aims to review, critique, and synthesize literature on a topic in an integrated way to stimulate new thinking, generate new perspectives, and encourage further research (Torraco, 2016). This paper draws on a systematic literature search undertaken for a previous study (reference blinded), that was updated for the current analysis.

### Systematic literature search

The initial search was conducted in January 2018 and focused on literature published since 2000. Electronic databases searched comprised: CINAHL, Embase, Medline, PsycINFO, ScienceDirect (Elsevier), and Sociology Source Ultimate. A manual search was conducted of specific journals not cataloged in these databases: *Addictive Behaviors* (2000 onward); *Asian Journal of Gambling Issues and Public Health* (2013 onward); *International Gambling Studies* (2001 onward); *International Journal of Mental Health and Addiction* (2006 onward); *Journal of Gambling Issues* (2008 onward); *Journal of Gambling Studies* (2000 onward); *Public Health* (2000 onward); and *Trauma, Violence and Abuse* (2000 onward). A combination of truncated search terms and Boolean logic was applied. Terms comprised: (Betting OR Wager* OR Gambl* OR Gaming*) AND (violen* OR victim* OR perpetrat* OR stalk* OR threat* OR abus* OR neglect* OR fight* OR haras* OR conflict* OR assault* OR aggress* OR batter* OR trauma* OR offen* OR murder* OR kill* OR homicide* OR rape* OR coerc* OR depriv* OR incest*). Search results were filtered to journal articles, abstract/title/keywords, full text availability, English language, 2000-18 publications, relevant subjects, and references available. The search results were stored and managed using Endnote X8.2.

**FIGURE 1:**
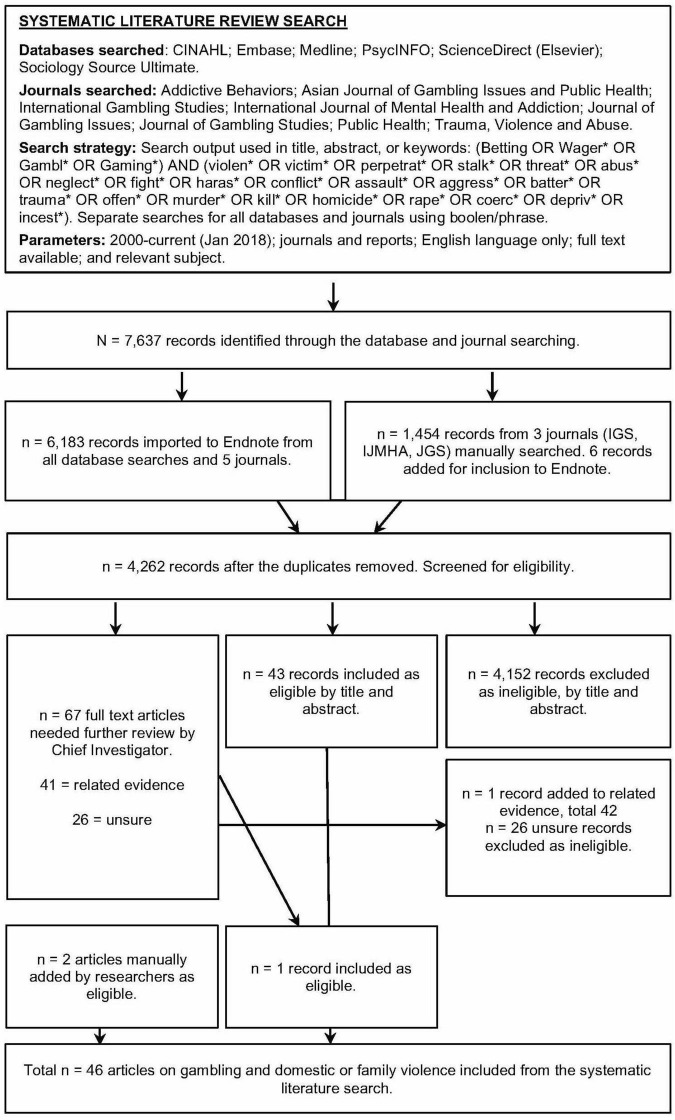
PRISMA flow diagram for the systematic literature review. Excluded = did not explore the relationship between gambling and DFV; Included = meets the inclusion criteria; Related evidence = did not explore the relationship between gambiling and DFV but includes related material of potential use; Unsure = not enough information in the title and abstract; full text article was examined.

Articles were eligible for inclusion if: (1) they provided quantitative and/or qualitative evidence of the co-occurrence of gambling problems and DFV/IPV; (2) the sample comprised adults, adolescents, or children recruited from any source; (3) the full-text was available in English; and (4) they were reported in a complete manuscript outlining original work published in a peer-reviewed journal from 2000 to January 2018. The search yielded 7,637 records, of which 6,189 were added to the Endnote library. After removing duplicates (*n* = 1,927), the inclusion criteria were then applied to screen the remaining 4,262 records. Those that did not meet the inclusion criteria were removed. In total, 46 journal articles met the inclusion criteria. Figure 1 displays the PRISMA flow diagram of the multi-step process of inclusion and exclusion of records.

### Targeted literature review

A targeted literature search was also conducted to capture gray literature which may not have been found in the systematic search. Websites for government, academic, and non-for-profit organizations related to gambling, domestic and family violence, and other related topics were searched. These were: Australia’s National Research Organization for Women’s Safety (ANROWS), Australian Institute of Family Studies (AIFS), Alberta Gambling Research Institute (AGRI), Gambling Research Australia (GRA), Gambling Research Exchange Ontario (GREO), and the Victorian Responsible Gambling Foundation (VRGF). Each source was searched using the same search terms and Boolean logic as used for the systematic search, and screened to full-text available, English language publications since 2000. A total of 30 eligible records were found.

### Updating

Prior to completing the current review, we updated the records to include 24 empirical studies, published as articles in any academic journal or in research reports since completing the 2018 search, indicating substantial recent growth in research specifically on gambling and DFV/IPV.

### Analytical approach

The extracted publications were examined to identify key themes, concepts, and findings, which enabled their classification into the categories in the sub-sections below. The review also drew on some literature outside the scope of the inclusion criteria to present concepts and findings that are relevant, but not specific, to gambling-related DFV/IPV – for example, literature related to DFV/IPV or to gambling, but not to both. This analysis enabled us to identify gaps in the literature and generate new perspectives to help guide future research.

## DFV/IPV linked to a perpetrator’s gambling

### Prevalence of DFV/IPV by perpetrators with a gambling problem

Several studies provide insights into the prevalence of DFV and IPV perpetrated by individuals with a gambling problem, but results vary across countries, samples and types of abuse examined. In convenience samples of treatment-seekers experiencing problem gambling, DFV perpetration rates in Australia have ranged from 19% (Dowling et al., 2021), to 23% (Dowling et al., 2014) to 49% (Lavis et al., 2015). New Zealand studies have found rates of 42% (Palmer du Preez et al., 2018) and 43% (Bellringer et al., 2017). In the United Kingdom, 12% of gambling treatment-seekers reported perpetrating IPV (Roberts et al., 2020). In a convenience sample of Gam-Anon members, 50% reported that their spouse with a gambling problem had physically or verbally abused them (Lorenz and Shuttlesworth, 1983). Also in a convenience sample, 56% of Canadian adults with a gambling problem reported using physical assault, injury, and/or sexual coercion against an intimate partner in the past year (Korman et al., 2008).

Population studies provide more representative findings. In Australia, 19.7% of problem/moderate risk gamblers and 19.3% of low-risk gamblers reported perpetrating DFV in the past 12 months, compared to 9.0% of non-problem gamblers (Dowling et al., 2018). Population surveys have also measured IPV perpetration by individuals with a gambling problem, but only in relation to physical violence. Estimates include 23% for lifetime perpetration in Edmonton Canada (Bland et al., 1993), 9% for past-5-year perpetration among United Kingdom males (Roberts et al., 2016), and 19% for females and 12% for males for past-year perpetration in the United States (Roberts et al., 2018). A meta-analysis of 14 studies estimated that 37% of people with a gambling problem have perpetrated physical IPV (Dowling N. et al., 2016).

Representative studies indicate increased odds of DFV/IPV perpetration amongst people with a gambling problem, although these estimates and their confidence intervals vary widely. In Australia, problem/moderate risk and low risk gamblers were 2.6–2.7 times more likely than non-problem gamblers to perpetrate DFV in the past-year (Dowling et al., 2018). In United States research, pathological gambling was associated with a fourfold increase in perpetrating severe physical dating violence and a 20-fold increase for severe physical marital violence (Afifi et al., 2010). Similar to Dowling et al.’s results for DFV (2018), IPV perpetration increased not only with pathological gambling, but also with milder gambling problems. A large United States survey found that having a gambling problem tripled the likelihood of perpetrating physical IPV (Roberts et al., 2018). A representative study with Lebanese women found that both physical and non-physical violence significantly increased if the perpetrator had a gambling addiction (Rahme et al., 2021). Non-representative studies also indicate that the odds of experiencing past-year IPV substantially increase if a partner has a gambling problem (Muelleman et al., 2002; Liao, 2008). A study with pregnant women in Vietnam found elevated odds of repeated IPV perpetration with *any* gambling by their husband (OR = 3.6; Nguyen et al., 2021).

Heightened rates of problem gambling are also apparent amongst DFV and IPV perpetrators (Korman et al., 2007; Afifi et al., 2010; Brasfield et al., 2011, 2012; Dowling et al., 2014; Izmirli et al., 2014). A meta-analysis estimated that 11% of perpetrators of physical IPV report problem gambling (Dowling N. et al., 2016), compared to an average of 2.3% of the general adult population who report problem gambling across numerous countries (Williams et al., 2012). Crime statistics also provide evidence of DFV/IPV linked to a perpetrator’s gambling but include only violent episodes recorded by police (Smith et al., 2003; Wheeler et al., 2010; Kuoppamäki et al., 2014).

### Characteristics of IPV perpetrators with a gambling problem

Research examining the psychological characteristics of IPV perpetrators with a gambling problem has yielded inconsistent results. Some studies have found gambling problems are uniquely associated with IPV after adjusting for other psychological factors. In a representative sample of United Kingdom men, problem and pathological gambling uniquely predicted several types of physical IPV when controlling for alcohol and drug dependence, mental disorder, and impulsivity (Roberts et al., 2016). Amongst male IPV perpetrators in a behavior change program, problem gambling was also uniquely associated with sexual aggression above and beyond alcohol use, impulsivity, and relationship satisfaction (Brasfield et al., 2012). Lavis et al. (2015) found no significant differences for alcohol misuse between treatment-seeking gamblers who had and had not perpetrated IPV.

In contrast, a large representative United States survey found that associations between problem gambling and physical IPV became non-significant when controlling for other psychological disorders (Roberts et al., 2018). Instead, alcohol abuse and personality disorders were significant predictors of physical IPV perpetration among males, while mood, anxiety, substance use, and personality disorders predicted IPV perpetration among females. Afifi et al. (2010) found similar results. Associations between IPV and both problem and pathological gambling became non-significant when controlling for the presence of a mental disorder. Alcohol use has been particularly implicated in the risk of IPV perpetration by individuals with a gambling problem (Dowling N. et al., 2016). Amongst emergency department patients, women with a partner with a gambling problem were 10 times more likely to have experienced IPV, and this risk increased to 50 times if the perpetrator also had a drinking problem (Muelleman et al., 2002). Clinical anger problems have also been implicated (Dowling N. et al., 2016). In one study, IPV was perpetrated by 74% of individuals with a gambling problem who also had anger problems, compared to 45% of those without anger problems (Korman et al., 2008). IPV perpetration was further elevated amongst those with both anger problems and a lifetime substance use disorder.

Mixed results have also been found in relation to the demographic characteristics of gamblers who perpetrate IPV. One review concluded that younger age and less than full employment significantly strengthened the relationship between problem gambling and IPV perpetration (Dowling N. et al., 2016). Conversely, in two representative population studies, the relationship between gambling and physical IPV remained significant after controlling for the perpetrator’s gender, age, relationship status, education, income, and ethnicity (Afifi et al., 2010; Roberts et al., 2018).

The above findings suggest a complex relationship between gambling problems and the characteristics of IPV perpetrators. Causal links have not been established. Individuals with a gambling problem and IPV perpetrators may simply share certain characteristics, such as a greater likelihood of mental health and substance use disorders. Alternatively, psychological disorders may interact with gambling problems to compound the likelihood of perpetrating IPV. A third-variable explanation is also possible.

### Explanations for links between gambling and DFV/IPV perpetration

Researchers have advanced several explanations for the elevated rates of DFV/IPV perpetration among people with a gambling problem. These explanations vary according to whether the gambling is thought to precede and trigger the violence, interact with other co-morbidities to increase the likelihood of violence, or exacerbate existing patterns of DFV/IPV. These explanations therefore differ in the causal role they assign to gambling in the perpetration of violence.

Where the development of a gambling problem is thought to precede perpetration, researchers have suggested that gambling losses fuel the gambler’s anger and frustration, as well as relationship conflict, which can trigger violent incidents (Muelleman et al., 2002; Korman et al., 2008; Afifi et al., 2010; MacLean et al., 2019). While cross-sectional surveys cannot determine temporal sequence, a qualitative analysis reported that help service clients abused by a family member who gambled attributed the violent episodes to their anger and irritation over gambling losses and subsequent arguments about family finances (Suomi et al., 2013). Research with Australian Aboriginal people reached similar conclusions (MacLean et al., 2019).

Stress created by gambling problems is a further possible explanation for why gambling might lead to violent acts. Financial stress typically increases with escalating gambling losses, depleting funds for household necessities, and sometimes leading to the sale of family assets and increased debt (Patford, 2009; Browne et al., 2016; Heiskanen, 2017). Gamblers can also face emotional stresses arising from financial and relationship tensions, and can feel ashamed, hopeless, and guilty about their gambling (Saugeres et al., 2012; Hing et al., 2020). Financial pressures, the gambler’s decreased attention to family responsibilities, and disruption to family functioning create relationship stress which may fuel arguments and violence (Grant Kalischuk et al., 2006; Patford, 2008, 2009; Holdsworth et al., 2013; Suomi et al., 2013; Klevan et al., 2019). High levels of stress in relationships are known to increase the likelihood of IPV (Capaldi et al., 2012).

Other research has identified the behavioral drivers of gambling disorder as exacerbating, but not necessarily preceding IPV. A study conducted for Australia’s National Research Organization for Women’s Safety (ANROWS) conducted interviews with 72 women abused by a male partner in relation to his or her gambling, and with 39 service providers in gambling help, DFV and crisis support services (Hing et al., 2020). Women victimized by a gambling partner described how his preoccupation with gambling overrode the importance he gave to other aspects of life, including the family’s welfare (Hing et al., 2022a,b). Reflecting the strong gambling urges and withdrawal symptoms that characterize problem gambling, male partners were reported to direct their anger, frustration, and blame at their partner when unable to gamble. Interviewees described cycles of violence connected to gambling events and losses, with tension building during periods of non-gambling that then exploded in violent acts. Threats to being able to continue gambling, such as the woman confronting her partner or lack of funds for gambling, were often met with violence. Many women described how the severity of both their partner’s gambling and his violence intensified over time.

Gambling may also interact with other co-morbidities to increase the likelihood of violence. IPV perpetration may be elevated amongst people with a gambling problem because of some shared psychological characteristics, such as anger problems, substance misuse, and mental disorders (Cunningham-Williams et al., 2007; Korman et al., 2008; Afifi et al., 2010). It is possible that gambling problems and DFV/IPV perpetration might co-occur, with the gambling having no influence on the violence. However, it is more likely that gambling exacerbates the violence even if the latter is primarily driven by other psychological co-morbidities. Substance use, in particular, is known to exacerbate IPV by weakening pro-social behavior by perpetrators (Muelleman et al., 2002; Korman et al., 2008; Brasfield et al., 2012; Noonan et al., 2017). In the ANROWS study (Hing et al., 2020, 2022b), several women reported that their partner’s gambling and gambling losses increased dramatically when he was affected by alcohol or certain drugs, especially “ice” (crystal methamphetamine), with violent episodes escalating quickly and viciously following these losses. Substance use was said to increase both the perpetrator’s gambling and the likelihood and severity of his subsequent violence. However, as noted earlier, the role of perpetrator characteristics in gambling-related IPV is unclear. The relationship between perpetration, problematic gambling, substance use, and other psychological characteristics of perpetrators is likely to be complex.

There is now widespread recognition that male partner violence against women is more likely to occur in contexts of gender inequality). Accordingly, a further explanation is that gambling can intensify DFV/IPV where gendered drivers of violence against women are present in a relationship and manifest as coercive and controlling behaviors (Hing et al., 2020, 2022a; Banks and Waters, 2022). This explanation sees gambling not as a cause of DFV/IPV, but a reinforcing factor that can exacerbate its frequency and severity. In the ANROWS study (Hing et al., 2020, 2022a), women described five main expressions of gender inequality in male partners that have been found to predict higher rates of IPV (Flood and Pease, 2006; Garcia-Moreno et al., 2006; Graham-Kevan and Archer, 2008; Heise, 2011). These included rigid and hierarchical gender expectations, condoning violent behavior against women in general, maintaining power and control in the relationship, restricting the woman’s autonomy, and valuing relationships with others who condoned disrespect toward women. Where these gendered drivers were present, male partners prioritized their gambling, controlled the family’s finances, coerced the woman into providing gambling funds, restricted her use of resources, and used violence to vent their anger, frustration, and blame. A qualitative study with Asian ethnic subgroups in New Zealand also explained IPV perpetration as an expression of the gambler’s desire to exert some control over their own and their family’s lives (Sobrun-Maharaj et al., 2012). In the United Kingdom, interviews with 26 women found that their male partner with a gambling problem often used instrumental coercive and controlling behaviors to access money for gambling, hide their gambling behavior, and blame the woman for their disordered gambling and abusive behavior (Banks and Waters, 2022).

Overall, the literature identifies several possible explanations for DFV/IPV perpetration by individuals experiencing a gambling problem. Importantly, not all people with a gambling problem perpetrate DFV/IPV, so gambling cannot be the sole cause of this violence. Instead, the violence is likely to result from a combination of factors that interact with the gambling. These factors may vary with the type of violence perpetrated. For example, situational violence may co-occur with the frustration, anger, stress, and conflict caused by a perpetrator’s gambling. Where gendered drivers of violence are also present, the stress caused by the gambling problem, along with its behavioral drivers, are likely to escalate the frequency and severity of violent behavior within an existing pattern of coercive control, particularly male partner violence against women. As such, the temporal sequence of gambling and DFV/IPV may also vary. A gambling problem may precede and increase the likelihood of perpetrating situational violence. However, a pattern of coercive and controlling behaviors may precede the gambling, which then exacerbates these behaviors to increase the frequency and severity of DFV/IPV.

## DFV/IPV linked to a victim’s gambling

### Prevalence of DFV/IPV against victims with a gambling problem

Having a gambling problem increases the likelihood of being a victim of DFV/IPV, particularly for women. This pattern has been found in help service, community, and population samples. In Australia, 27% of a sample attending gambling help services reported past-year victimization involving physical DFV; and this rate was significantly higher amongst women (38%) than men (21%) (Dowling et al., 2014). In another Australian study of gambling treatment-seekers, 20% reported DFV victimization, and this rate was again higher amongst women (Lavis et al., 2015). In New Zealand, the past-year DFV victimization rate in a sample of gambling treatment-seekers was 57% for women compared to 44% for men, averaging 49% overall (Bellringer et al., 2017). Another New Zealand study found a similar victimization rate of 47%, which again was higher amongst female (42%) than male gamblers (28%) (Palmer du Preez et al., 2018). Gamblers were most commonly victimized by a current or former intimate partner (Dowling et al., 2014; Bellringer et al., 2017; Palmer du Preez et al., 2018). In Spain, 69% of women recruited from gambling treatment services reported recent IPV victimization, a 10-fold higher rate than found among women in the general population (Echeburúa et al., 2011). In Canada, 49% of a convenience sample of individuals with a gambling problem reported being victims of past-year physical IPV, and this was elevated amongst women (Korman et al., 2008).

Representative studies have also found higher prevalence of DFV/IPV victimization amongst people with a gambling problem. In Australia, 21.3% of problem/moderate risk gamblers and 20.0% of low risk gamblers reported past-year DFV victimization, compared to 9.4% of non-problem gamblers (Dowling et al., 2018). A representative United States study (Afifi et al., 2010) found that being a victim of “minor physical dating violence” and of “severe physical marital violence” was associated with increased odds of pathological gambling, after adjusting for socio-demographic factors and mental disorders. A representative prospective United States study measured past-year problem gambling and other psychiatric disorders at baseline, and past-year physical IPV 3 years later (Roberts et al., 2018). After controlling for socio-demographic variables, problem gambling was associated with a threefold increase in the odds of physical IPV victimization, but only amongst women.

In contrast, two studies have found no association between gambling problems and DFV victimization. One was constrained by the small sub-sample with a gambling problem and use of non-standard problem gambling measures (Schluter et al., 2008); the other included only clients of a residential substance use service (Rudd and Thomas, 2016). With these two exceptions, research has consistently found that problem gambling is positively associated with DFV/IPV victimization.

### Characteristics of DFV/IPV victims with a gambling problem

DFV/IPV victims with a gambling problem are significantly more likely to be women. However, after controlling for gender, as well as other sociodemographic characteristics including age, relationship status, education, income, and ethnicity, population studies indicate that gambling problems are still significantly associated with being a victim of physical IPV (Afifi et al., 2010; Dowling et al., 2018; Roberts et al., 2018). One study found this relationship was especially strong at high levels of gambling severity among females (Roberts et al., 2018), while three studies have found this relationship was also significant for less severe gambling problems (Afifi et al., 2010; Dowling et al., 2018; Roberts et al., 2018). Overall, these studies indicate that the presence of a gambling problem is more important than a victim’s sociodemographic characteristics in explaining victimization.

Some psychological characteristics of victims may attenuate the relationship between gambling problems and DFV/IPV victimization, although results are mixed. Afifi et al. (2010) found that associations between experiencing a gambling problem and being a victim of some types of physical violence remained significant after adjusting for lifetime presence of a mental disorder. Conversely, Roberts et al. (2018) found that the relationship between gambling and IPV victimization was no longer significant when controlling for co-occurring psychiatric disorders. Specifically, alcohol abuse, drug use, and personality disorders were significant predictors of physical IPV victimization among males, while mood, anxiety, alcohol abuse, and personality disorders were associated with victimization among females. In Dowling et al.’s study (2018), the relationship between DFV victimization and problem/moderate risk gambling amongst victims became non-significant after adjusting for demographics, substance use, and psychological distress. Nonetheless, the relationship between DFV victimization and low risk gambling remained significant after controlling for these factors. Overall, psychological comorbidities may contribute to understanding relationships between problem gambling and victimization, but any causal directions are unclear. While it is important to understand who might be more vulnerable to DFV/IPV victimization, a victim’s characteristics cannot explain this violence given that it is perpetrators who choose to use violence. It is also important to recognize that psychological comorbidities may result from DFV/IPV victimization, where victims gamble to cope with the stress and psychological trauma caused by their abuse.

### Explanations for links between gambling and DFV/IPV victimization

Researchers have advanced several explanations for the elevated rates of DFV/IPV victimization among people with a gambling problem. These explanations vary according to whether the gambling is thought to precede and trigger victimization, or whether the gambling occurs as a response to victimization.

Problem gambling has been reported to precede and be a cause of victimization due to the domestic conflict associated with the financial and other stresses caused by gambling (Korman et al., 2008). In Suomi et al.’s (2013) study, the 11 interviewees who had perpetrated violence against a family member reported that their violence was fueled by their accumulated anger and mistrust arising from the victim’s gambling. However, perpetrators’ explanations for their violence are unreliable, since they frequently use victim-blaming and external “causes” to justify their violence (Henning and Holdford, 2006), including blaming the victim’s gambling (Browne et al., 2016; Hing et al., 2020; O’Mullan et al., 2022a). Nevertheless, problem gambling undoubtedly has deleterious effects on families. These include depleted finances; anger, arguments, and conflict; mistrust, lies and deception; relationship dissatisfaction; neglect of family and family responsibilities; poor family functioning; and development of gambling or other addictions within the family (Grant Kalischuk et al., 2006; Dowling N. A. et al., 2016). These impacts may increase the likelihood of violence and abuse by family members.

Intimate partners are the most severely affected. They typically feel shocked to learn of the gambling, followed by feelings of betrayal, anger, despair, and fear, and ongoing anxiety, depression, and emotional exhaustion (Hodgins et al., 2007; Shaw et al., 2007; Patford, 2008, 2009; Grant Kalischuk, 2010; Valentine and Hughes, 2010; Holdsworth et al., 2013). When a gambling problem is revealed, financial stress is often already acute, since the problem is not usually disclosed until crisis-point (Suurvali et al., 2010; Hing et al., 2012; Hing and Russell, 2017a,b). Problem disclosure may reveal substantial debt, loss of lifetime savings, or the need to sell the family home (Holdsworth et al., 2013). This financial devastation and the realization of their partner’s prolonged deceit typically cause significant distress. Partners may then have to deal with numerous cycles of continued gambling, quit attempts, and relapse. Since relapse is common (Battersby et al., 2010), the gambling problem may persist over many years. Partners therefore experience prolonged stress, resulting in accumulated anger, mistrust, and conflict which can increase the risk of violence (Suissa, 2005; Kourgiantakis et al., 2013; Suomi et al., 2013).

Where gambling precedes the violence, these accumulated tensions and emotions may trigger situational violence in direct response to the victim’s gambling (Korman et al., 2008; Suomi et al., 2013, 2019). However, the ANROWS study (Hing et al., 2020, 2022d; O’Mullan et al., 2022a) found that only a minority of the women interviewed first experienced IPV after they started gambling. Further, their subsequent victimization was not always limited to violent incidents that were an immediate response to their gambling, although these continued to occur. Instead, abusive behavior came to permeate the relationship and manifested as ongoing denigration, disrespect, and violence. A more common pattern was for these violent incidents to occur in contexts of coercive control. That is, the victim’s gambling was reported to be used by the perpetrator as an excuse to intensify the violence already occurring in the abusive relationship.

Gambling can also be a response to violence, reflected in high rates of victimization amongst people with a gambling problem (Kausch et al., 2006; Afifi et al., 2010; Hodgins et al., 2010; Nixon et al., 2013a,b; Andronicos et al., 2015). Amongst adults abused as children, this victimization clearly precedes the gambling problem. Gambling can also be used to cope with violence-induced trauma experienced in adulthood. Amongst 212 clients of gambling help services, Suomi et al. (2019) observed this pattern only amongst women, and suggested they had likely experienced severe traumatic abuse linked to a pattern of coercive and controlling behaviors by their partner. Women in other studies have described gambling to cope with an abusive intimate relationship; and in some cases, they faced more violence after their subsequent gambling losses (Saugeres et al., 2014; Centre for Innovative Justice, 2017).

Recent findings are consistent with this earlier research. In the ANROWS study (Hing et al., 2020, 2022d; O’Mullan et al., 2022a), most women reported frequently experiencing severe and chronic violence from their partner, before they commenced gambling. These women reported gambling to gain physical escape from the violence, or psychological escape to cope with the resultant trauma, in a quest to regain some control over their lives, and to cope with the legacy of past abuse after separating from a violent partner (Hing et al., 2022d). Other studies report that relationship difficulties, including family violence, are life events that can lead women into harmful gambling behaviors (Granero et al., 2022; McCarthy et al., 2022). Women are more likely than men to gamble for avoidance-based coping, and to seek respite from abusive partners, difficult relationships, social isolation, emotional pain, and worries (Grant and Kim, 2002; Thomas and Moore, 2003; Lloyd et al., 2010; Saugeres et al., 2012).

Women are particularly drawn to electronic gaming machines (EGMs), whose structural characteristics facilitate dissociation and time-out from a difficult reality and pose substantial risk of persistence and dependency (Dowling et al., 2005; Livingstone, 2005; Schüll, 2005, 2012). In addition, gambling venues can be a refuge for escape, since they often provide highly accessible, comfortable, and female-friendly environments that have good security and long opening hours (Brown and Coventry, 1997; Surgey, 2000; Thomas et al., 2009, 2011a,2011b; Rintoul et al., 2017; Hing et al., 2017). In the ANROWS study, women escaping IPV reported being attracted to venues because of their social, geographic, and temporal accessibility, allowance for uninterrupted play on EGMs, and the addictive nature of EGMs (Hing et al., 2020, 2022d; O’Mullan et al., 2022a). The push factors that drove these women to try to escape their abuse, and the pull factors that attracted them to gambling venues, led to these women’s gambling problem. Their partner’s violence typically escalated with the gambling, since it provided additional stress, conflict, and excuses to perpetrate further violence and exert controlling behaviors to restrict her gambling. The relationship between these women’s gambling and their victimization tended to be cyclical, self-reinforcing, and characterized by an escalation of both issues over time.

In summary, gambling has been viewed as both a cause and a consequence of DFV/IPV victimization. However, most partners and family members of people with a gambling problem do not act violently toward them, so problem gambling and related stressors cannot be a sole cause of victimization. Instead, DFV/IPV is likely to co-occur with a combination of factors that interact with the victim’s gambling, as well as the perpetrator’s characteristics, behaviors, and choices. These factors may vary with the type of violence perpetrated. For example, gambling may precede the DFV/IPV and result in situational violence triggered by the anger, mistrust, and conflict that arise from the victim’s gambling. Where a pattern of coercive control already exists, gambling-related stress is likely to escalate the frequency and severity of violent behavior. Being a victim of violence can also lead to the commencement or escalation of gambling to cope with the resultant trauma, and as a means of physically escaping a violent household. This appears to particularly occur amongst women subjected to chronic violence who use EGM gambling as a means of avoidance-based coping and gambling venues as “safe” refuges. However, their gambling can compound their victimization by providing perpetrators with an “excuse” to use violence and subject them to further control.

## Discussion

Previous research has drawn much needed attention to the co-occurrence of gambling problems and DFV/IPV. Strengths include results based on large population samples in several countries, comparative analyses of DFV/IPV among victims and perpetrators with and without gambling problems, and rich insights from qualitative studies. However, research efforts to date also have some limitations that may mask a more accurate understanding of gambling and DFV/IPV, and which can highlight potential future improvements in this research area. We discuss these below to encourage research approaches that can continue to advance understanding to better inform future research, policy, and practice.

### Limitations of focusing on situational violence

Quantitative studies have used acts-based instruments that focus on situational violence to measure DFV/IPV perpetration and victimization amongst people experiencing a gambling problem. These have included the Hurt-Insult-Threaten-Scream instrument (HITS; Sherin et al., 1998), the Conflict Tactics Scale-2 (CTS2; Straus et al., 1996), and the Jellinek–Inventory for Assessing Partner Violence (JIPV; Kraanen et al., 2013). The HITS instrument asks about causing or being (1) physically hurt, (2) insulted or talked down to, (3) threatened with harm, and (4) screamed or cursed at, in relation to a family member/partner. The CTS2 asks how often the respondent has perpetrated or been a victim of 39 behaviors in the domains of negotiation, psychological aggression, physical assault, sexual coercion, and injury. However, population gambling studies have administered only the six items relating to physical aggression. The JIPV asks about perpetration and victimization involving two behaviors—threatening behaviors and physically abusive behaviors. Gambling studies have usually administered these instruments to ask whether each behavior occurred in the last 12 months.

Numerous researchers have criticized these types of acts-based measures for narrowly conceptualizing and measuring DFV as individual occurrences of violent acts (e.g., Dobash et al., 1992; Kimmel, 2002; Rabin et al., 2009; Flood, 2010; Bender, 2017; Loveland and Raghavan, 2017; Crossman and Hardesty, 2018). These measures of situational violence prioritize tallying the number, types, and odds of experiencing violence within certain researcher-defined parameters, over understanding a complex phenomenon as defined by the victim (Bender, 2017). For example, what a victim feels to be emotional abusive may differ from how a measure defines it; and while physical violence is typically perceived as more severe than psychological abuse (Stark and Hester, 2019), a victim may experience ongoing psychological torment as much more damaging than an occasional slap. Experiences of violence vary by type, severity, directionality, and whether they are chronic or episodic (Bogat et al., 2005), but these variations have not been captured well in measures used in gambling research. This is especially the case where measures combine types of DFV/IPV into one or a few items, and do not consider the frequency and severity of violence, violent acts committed in self-defense, and the accumulated impacts of chronic abuse. While studies to date have focused attention on gambling and DFV/IPV, it is important to recognize the limitations of the measures used and how these may have affected prevalence estimates, analyses of personal characteristics associated with perpetration and victimization, and explanations for the phenomenon.

### The need to also measure coercive control

Gambling studies have not yet attempted to measure coercive control. Quantifying if any abuse has occurred, or the number and types of abusive incidents, does not capture this systemic pattern of control and coercion that characterizes much of the violence against women (Bender, 2017), and can also be experienced by men (Morgan and Wells, 2016; Bates, 2020; Walker et al., 2020). Current measures also do not capture victims’ associated experiences, such as ongoing fear, hypervigilance, accumulated trauma, enforced isolation, restricted autonomy, and lack of control in their lives, relationships, activities, and decision-making. Nonetheless, while acts-based measures are not designed to measure coercive control, a few of the questions in some instruments may capture some aspects of this “intimate terrorism” (Johnson, 2005, 2010). Some researchers have therefore reanalyzed responses to items that appear to reflect abuse that is ongoing, denigrating, perceived as threatening, and causing fear, such as “Repeatedly belittled you to the extent that you felt worthless” and “Frightened you, by threatening to hurt you or someone close to you” (Myhill, 2015). Myhill’s (2015) analysis, and another study that also reanalyzed national data (Johnson et al., 2014), found that women were 4–5 times more likely than men to be victims of coercive and controlling behaviors from their partner, while perpetration of situational violence showed greater parity between men and women. Further, coercive controlling violence was accompanied by more severe, injurious, and frequent acts of violence, as well as the victim’s psychological distress, and was more likely to persist over time, compared to situational violence. These are important differences which can inform the allocation of resources and services to help prevent DFV/IPV and assist victims. Where possible, similar reanalyzes of DFV/IPV data from previous population gambling studies are likely to yield insights that are also more nuanced.

Given the stark differences in the nature and experiences of intimate terrorism compared to situational violence, future gambling studies of DFV/IPV should also measure coercive control. Over 20 different measures of coercive control have been used in DFV/IPV studies; they focus on varying aspects of the behavior, and there is currently no gold standard instrument (Hamberger et al., 2017). This presents a challenge for future studies to determine the best measure, or combination of measures, for use in gambling research. Nonetheless, DFV/IPV research has shifted toward investigating violent behaviors that are ongoing rather than incident-based, including controlling and isolating behaviors, as well as technology-assisted monitoring, and stalking. Gambling research would also benefit from a greater focus on patterns of ongoing violence and not just acts of violence, to gain a more complete picture of DFV/IPV.

### A need to consider gender differences in experiences of violence

Measuring only situational violence ignores the world beyond the dataset (Reed et al., 2010), obscuring patterns of violence that have qualitative and quantitative gender differences in terms of extent, severity, intentions, motivations, and impacts (Dobash et al., 1992; Rabin et al., 2009; Flood, 2010). Women’s violence is more likely to be reactive, retaliatory, and committed in self-defense, and less likely to be lethal or result in serious injury; while men tend to use violence more instrumentally and injuriously to control women’s lives (Archer, 2000; Kimmel, 2002; Johnson, 2006). Victims’ experiences of violence also vary by gender. Gendered drivers of violence by men against women were discussed earlier. Patriarchal structures and traditional gender roles also affect men’s experiences of violence. Social expectations to conform to masculine norms can be reflected in emasculating and homophobic psychological abuse of men, failure to acknowledge their experiences as violence, and their reluctance to disclose the abuse and seek help (Walker et al., 2020; Scott-Storey et al., 2022). Experiences of coercive control also vary. For example, men tend to control a female partner through threatened or actual physical violence, while creating fear of degradation, public humiliation, and losing access to their children are more often used to control men (Hines et al., 2007; Nybergh et al., 2016; Bates, 2020). Experiences of violence also vary in same-sex relationships (Carvalho et al., 2011; Oliffe et al., 2014), but these have not yet been examined in gambling studies.

Gender differences in DFV/IPV are further obscured where measures ask only whether or not a violent act has occurred, and do not assess the types, frequency, severity, or impacts of the violence. Administering acts-based measures such as the Conflict Tactics Scale (CTS; Straus, 1990) does not adequately capture gender differences in experiences of violence. Especially measuring coercive control, including but not limited to the subforms mentioned above (i.e., threatened or actual physical violence, degradation, public humiliation, threat of losing access to children), could give a more nuanced picture of the forms of violence each gender is faced with, resulting in more specialized forms of help. The limitation of using the CTS in most studies is not specific to gambling research but applies to the measurement of DFV/IPV more broadly. Overall, DFV/IPV is underreported by both men and women, but their reasons for this vary (Felson et al., 2002; Birdsey and Snowball, 2013).

Greater sensitivity to gender differences in experiences of DFV/IPV, that is reflected in research approaches and instruments, would enable gambling research to provide more accurate and nuanced findings. A gender-based perspective may help to shift gambling research beyond its predominantly situational violence perspective that assumes that all DFV/IPV arises from escalating conflicts in relationships, to also recognize the use of power and control in violent relationships (Bender, 2017). This shift is evident in some qualitative gambling research (Hing et al., 2021, 2022a,2022b,2022d,2022c; Banks and Waters, 2022; O’Mullan et al., 2022a), but not in the quantitative measures used. Gambling research should examine IPV perpetrated by men against women, by women against men, and in same-sex relationships, using approaches and instruments that are sensitive to different conceptualizations, experiences, and reporting of violence in these contexts.

While gender differences are important on the whole, the more pressing issue may be to give a more detailed picture of violence experienced by women. DFV/IPV research, therefore, has shifted toward using a wide range of data drawn from police, court, and corrections records, hospital, coroners’, and mortality reports, child protection and homelessness services, helplines and other support services, as well as personal safety surveys. In Australia, triangulation of these data provides clear evidence that DFV/IPV is more likely to be perpetrated by men against women and with more severe impacts, including a threefold higher risk of being killed by their partner [Cussen and Bryant, 2015; Cox, 2016; Australian Institute of Health and Welfare [AIHW], 2019]. Data collected and published by the Australian Government indicates that women are 2.7 times more likely to have experienced physical or sexual violence, 2.5 times more likely to have experienced stalking, and 1.5 times more likely to have experienced emotional abuse from a current or former partner (Australian Bureau of Statistics, 2017). Globally, based on its multi-country studies, the World Health Organization [WHO] (2012) concluded that “The overwhelming global burden of IPV is borne by women.” As discussed by Keating (2015), two interconnected themes appear to be particularly important in the context of gambling: the importance of control/dominance among male perpetrators, and the role of economic stress in relation to masculinity (Peralta and Tuttle, 2013). Below, we discuss how economic stress and economic abuse can interact with gambling-related IPV.

### A need to include economic abuse linked to gambling as a type of DFV/IPV

To our knowledge, no gambling surveys have measured economic abuse linked to gambling, even though qualitative research indicates that having a gambling problem provides a strong motivation for economic abuse (Patford, 2009; Banks and Waters, 2022; Hing et al., 2022c). Kutin et al. describe economic abuse as “behaviors aimed at manipulating a person’s access to finances, assets and decision-making to foster dependence and control” (2017, p. 1). Adams et al. (2008) identified two overarching behaviors involved in economic abuse: (1) economic control preventing resource acquisition and use, and (2) economic exploitation of the victim’s resources. Partners may be particularly vulnerable to economic abuse, since intimate partnerships usually involve shared bank accounts, a partner’s access to personal details for identification, opportunities for domestic theft and, sometimes, agreement that one partner will manage the finances (Lind et al., 2015). Where the partner managing the finances has a gambling problem, the risk of economic abuse appears to be high (Hing et al., 2022c).

Practitioners have been highlighting economic abuse linked to gambling for some time (Rabiner et al., 2005; Conrad et al., 2011; Bagshaw et al., 2013; Johannesen and LoGiudice, 2013; Kutin et al., 2019). Research into the financial impacts of gambling on partners has also discussed behaviors that constitute economic abuse, but has rarely framed them as DFV/IPV (Patford, 2009; Holdsworth et al., 2013). In a study of IPV, Hing et al., 2020, 2022b,2022c interviewed women subjected to economic abuse by a male partner with a gambling problem. Economic exploitation included the partner withdrawing money from her bank accounts without her knowledge; redraws on mortgages without permission; unauthorized sale of family property; coercing her into lending him money and taking on debt; stealing her property; and gambling all his income, household money, and family savings. Economic control occurred where the male partner retained complete control over the family’s finances and denied her access to money, including for household essentials. In an ongoing pattern of coercive control, physical and psychological violence was used to reinforce the economic abuse, as also found in a qualitative study by Banks and Waters (2022). These women experienced significant poverty and deprivation that trapped them in the relationship and extended their victimization. Older women described being drained of all assets they had accumulated over a lifetime of work, resulting in chronic poverty (Hing et al., 2021). Other research has highlighted these legacy effects on a partner’s financial wellbeing, including losing their home, being unable to ever purchase another home, diminished inheritance for their children, lack of child support payments, and ongoing pressure for money post-separation (Patford, 2009). A strong link is empirically established between being a victim of economic abuse and subsequent economic hardship (Adams et al., 2008). To our knowledge, no studies have examined economic abuse by female gamblers against their male partners, or in same-sex relationships.

Sustaining a gambling problem requires substantial amounts of money and causes significant financial stress, providing a strong motivation for economic abuse. Economic abuse involves exploitative and controlling behaviors that cause substantial harm to victims, and should therefore be included in studies of DFV/IPV that seek to establish its nature and prevalence. This is particularly the case in gambling studies, where gambling problems and economic abuse are likely to co-occur. Including economic abuse in future gambling studies would provide a more complete understanding of DFV/IPV perpetration and victimization linked to gambling.

### A need to consider contextual contributors to DFV/IPV linked to gambling

Minimal research has examined factors that contribute to DFV/IPV linked to gambling beyond individual and relationship factors pertaining to victims and perpetrators. One study considered the relationship between access to gambling and DFV. It found significant associations between police-recorded DFV and accessibility to EGMs at the postcode level in Victoria Australia (Markham et al., 2016). The ANROWS study (Hing et al., 2020, 2022a; O’Mullan et al., 2022a,b) explored how the gambling industry, police and justice systems, victim support services, financial institutions, and social norms can contribute to contexts that exacerbate this violence. In alignment with a public health perspective on gambling harm, research could valuably examine how gambling industry products, practices, environments, and marketing are contributing and responding to DFV/IPV. These are issues that are unlikely to be examined in the broader literature on DFV/IPV and therefore warrant attention from gambling researchers. Findings could then inform efforts to improve prevention, harm reduction, and support for victims of DFV/IPV when linked to gambling.

### Summary of the current focus and gaps in knowledge in gambling studies of DFV/IPV

**FIGURE 2:**
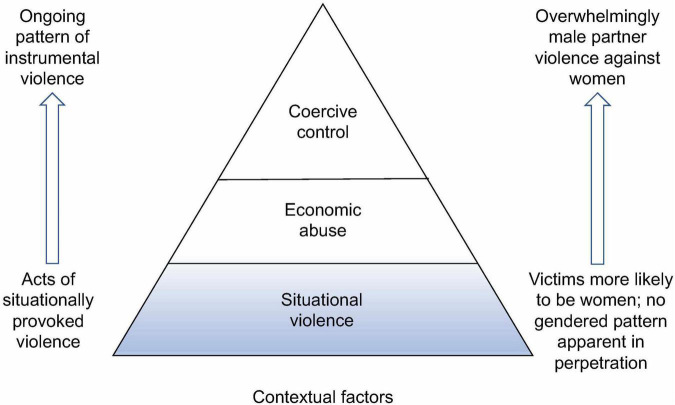
Current focus of gambling studies of DFV/IPV and gaps in areas of knowledge.

Figure 2 shows the current focus of gambling studies of DFV/IPV and gaps in areas of knowledge. The shaded area depicts the main focus to date, which has been on situational violence in response to gambling losses and tensions. The unshaded areas are those which have received very little research attention. Moving up the pyramid, patterns of instrumental violence are more likely to constitute male partner violence against women, in contrast to current research that indicates more gender parity in the perpetration of violence linked to gambling. Accordingly, gambling research currently provides only a partial picture of DFV/IPV linked to gambling. We hope that the additional perspectives provided in this paper will generate further research to help address the remaining gaps, particularly in relation to coercive control, economic abuse, chronic and gendered patterns of violence, and contributing contextual factors.

## Conclusion

This paper has reviewed research on the links between gambling and DFV/IPV, including prevalence, characteristics of victims and perpetrators, and explanations for gambling-related violence. Based on this review, the paper has suggested several potential improvements that can be considered in future studies. These include a shift from focusing on situational violence to also include coercive control, greater sensitivity to gender differences in experiences of violence in research design and interpretation, and the need to include economic abuse as a form of DFV/IPV. Adopting a public health lens to broaden the research focus from victims and perpetrators to also consider contextual factors would also be valuable. In particular, gambling research should examine the contribution of gambling products, practices, environments, and marketing to DFV/IPV and how this might be ameliorated. While research to date has drawn much needed attention to the relationships between gambling and DFV/IPV, we hope that our suggestions can be used to generate more complete, accurate, and nuanced findings to inform future policy and practice.

## Author contributions

NG conducted the initial literature search, which was subsequently updated by NH. NH wrote the first draft of the manuscript. CO’M, LM, HB, and NG refined the manuscript. All authors approved the submitted manuscript.
